# Supplementary material for: Metabolic Biomarkers in Adults with Type 2 Diabetes: The Role of PPAR-γ2 and PPAR-β/δ Polymorphisms
Source: Biomolecules. 2023 Dec 14;13(12):1791. doi: 10.3390/biom13121791 (PMC10741495; doi:10.3390/biom13121791)
Supplement: Supplementary file 1 [file biomolecules-13-01791-s001.zip › biomolecules-2738369-supplementary.pdf]

Supplementary Table S1. Anthropometric and biochemical measures by PPAR genotypes (codominant model).

Variable	PPAR-γ2 rs1801282				PPAR-β/δ rs2016520			
	CC (n=238) Md (IQR)	CG (n=67) Md (IQR)	GG (n=5) Md (IQR)	*p*	TT (n=231) Md (IQR)	TC (n=79) Md (IQR)	CC (n=4) Md (IQR)	*p*
BMI (kg/m^2)	31 (27-35)	32 (28-34)	29 (27-30)	0.540	31 (27-35)	32 (28-35)	31 (28-33)	0.423
Waist circumference (cm)	101 (95-110)	106 (99-115)	102 (90-112)	0.072	100 (95-110)	104 (100-115)	98 (94-112)	0.103
FPG (mg/dL)	144[a] (114-179)	157[b] (128-207)	106[ab] (102-217)	0.040	145 (117-177)	158 (124-199)	138 (131-145)	0.197
HbA1c (%)	7 (6-9)	8 (6-10)	8 (6-10)	0.157	7 (6-9)	8 (6-10)	6 (6-7)	0.063
TG (mg/dL)	177 (126-235)	178 (137-256)	218 (212-220)	0.490	174[a] (124-227)	195[b] (159-282)	148[ab] (110-232)	0.046
TC (mg/dL)	194 (164-220)	189 (167-216)	197 (194-239)	0.656	193 (165-217)	189 (163-226)	197 (170-242)	0.778
HDL-C (mg/dL)	44 (36-54)	46 (38-55)	51 (48-58)	0.186	44 (37-54)	47 (36-56)	39 (38-46)	0.548
LDL-C (mg/dL)	110 (80-134)	101 (73-129)	94 (91-144)	0.429	109 (79-132)	103 (64-139)	125 (94-165)	0.478

VLDL (mg/dL)	35 (25-47)	36 (27-51)	44 (42-44)	0.490	35[a] (25-45)	39[b] (32-56)	30[ab] (22-46)	0.046
TC/HDL-C index	4 (3-5)	4 (3-5)	4 (3-5)	0.465	4 (3-5)	4 (3-5)	5 (4-6)	0.716

BMI (body mass index), FPG (fasting plasma glucose), TG (triglycerides), TC (total cholesterol), HDL-C (high-density lipoprotein), LDL-C (low-density lipoprotein), VLDL (very-low-density lipoprotein). Different letters in superscripts indicate statistical difference by Dunn's test after Kruskal Wallis test ([a-b]).

Supplementary Table S2. Multivariate analysis of the relation between PPARs polymorphisms and fasting plasma glucose (log mg/dL).

	**Dominant Model**				**Over-dominant Model**			
	**PPAR-γ2 rs1801282 CC *vs.* CG + GG**				**PPAR-γ2 rs1801282 CC + GG *vs.* CG**			
	**Model 1** n=294	**Model 2** n=294	**PPAR-β/δ rs2016520**		**Model 1** n=294	**Model 2** n=294	**PPAR-β/δ rs2016520**	
			**TT** n=217	**TC + CC** n=77			**TT + CC** n=219	**TC** n=75
**Variable**	Beta $_{95\%}$CI *p*	Beta $_{95\%}$CI *p*	Beta $_{95\%}$CI *p*	Beta $_{95\%}$CI *p*	Beta $_{95\%}$CI *p*	Beta $_{95\%}$CI *p*	Beta $_{95\%}$CI *p*	Beta $_{95\%}$CI *p*
PPAR-γ2 rs1801282	0.13* (0.04, 0.23) 0.007	0.06 (-0.05, 0.17) 0.260	0.06 (-0.04, 0.17) 0.246	0.34 (0.14-0.54) 0.001	0.14+ (0.04, 0.24) 0.005	0.07 (-0.04, 0.19) 0.192	0.08 (-0.03, 0.19) 0.175	0.33 (0.13, 0.54) 0.002
PPAR-β/δ rs2016520	0.09# (0.00, 0.18) 0.060	0.03 (-0.07, 0.13) 0.587	--	--	0.09‡ (0.01, 0.18) 0.055	0.03 (-0.07, 0.14) 0.521	--	--
PPAR-β/δ X PPAR-γ2	--	0.28 (0.06, 0.50) 0.013	--		---	0.26 (0.04, 0.48) 0.020	--	--

Adj R^2	*0.05*	*0.07*	*0.01*	*0.17*	*0.06*	*0.07*	*0.01*	*0.17*

All models were adjusted for participants´ age at the time of T2D diagnosis. * Reference CC, [#] reference TT, [+] reference CC + GG, [‡] reference TT + C
